# Induction of KIAA1199/CEMIP is associated with colon cancer phenotype and poor patient survival

**DOI:** 10.18632/oncotarget.5921

**Published:** 2015-09-30

**Authors:** Stephen P. Fink, Lois L. Myeroff, Revital Kariv, Petra Platzer, Baozhong Xin, Debra Mikkola, Earl Lawrence, Nathan Morris, Arman Nosrati, James K. V. Willson, Joseph Willis, Martina Veigl, Jill S. Barnholtz-Sloan, Zhenghe Wang, Sanford D. Markowitz

**Affiliations:** ^1^ Department of Medicine, Case Western Reserve University, and Case Medical Center, Cleveland, OH, USA; ^2^ Department of Pathology, Case Western Reserve University, and Case Medical Center, Cleveland, OH, USA; ^3^ Department of Epidemiology and Biostatistics, Case Western Reserve University, and Case Medical Center, Cleveland, OH, USA; ^4^ Department of Genetics and Genome Sciences, Case Western Reserve University, and Case Medical Center, Cleveland, OH, USA; ^5^ Case Comprehensive Cancer Center, Case Western Reserve University, and Case Medical Center, Cleveland, OH, USA; ^6^ Simmons Comprehensive Cancer Center, University of Texas Southwestern Medical Center, Dallas, TX, USA; ^7^ Genomic Medicine Institute, Lerner Research Institute, Cleveland Clinic Foundation, Cleveland, OH, USA

**Keywords:** colon cancer, CEMIP, secreted protein, prognostic marker, metastasis

## Abstract

Genes induced in colon cancer provide novel candidate biomarkers of tumor phenotype and aggressiveness. We originally identified KIAA1199 (now officially called CEMIP) as a transcript highly induced in colon cancer: initially designating the transcript as Colon Cancer Secreted Protein 1. We molecularly characterized CEMIP expression both at the mRNA and protein level and found it is a secreted protein induced an average of 54-fold in colon cancer. Knockout of CEMIPreduced the ability of human colon cancer cells to form xenograft tumors in athymic mice. Tumors that did grow had increased deposition of hyaluronan, linking CEMIP participation in hyaluronan degradation to the modulation of tumor phenotype. We find CEMIP mRNA overexpression correlates with poorer patient survival. In stage III only (*n* = 31) or in combined stage II plus stage III colon cancer cases (*n* = 73), 5-year overall survival was significantly better (*p* = 0.004 and *p* = 0.0003, respectively) among patients with low CEMIP expressing tumors than those with high CEMIP expressing tumors. These results demonstrate that CEMIP directly facilitates colon tumor growth, and high CEMIP expression correlates with poor outcome in stage III and in stages II+III combined cohorts. We present CEMIP as a candidate prognostic marker for colon cancer and a potential therapeutic target.

## INTRODUCTION

Colon cancer is the second leading cause of cancer death among adult Americans, with an estimated 136,800 new cases and 50,300 deaths in 2014 [1]. If detected early, patients with localized, resectable disease have a favorable prognosis with a 91% 5-year overall survival rate, yet survival rates decline significantly with disease progression [2]. Patients with metastatic disease to distant organs at the time of presentation have an expected 5-year overall survival rate of only 12% and a median survival time of 29 months [3]. Despite improved understanding of the molecular events leading to colon cancer, there is a continued need for prognostic markers that can predict disease progression, metastasis, or recurrence in patients with the disease. For example, patients with stage II colon cancer, in which overall survival is already ∼80% with surgery alone, the benefit of adjuvant therapy does not improve survival by more than 5%, thus underscoring the need for better prognostic markers to determine low from high risk stage II patients [4]. Additionally, elucidation of genes necessary for the metastatic process can lead to new avenues for targeted therapies.

Genes induced in colon cancer provide novel candidate biomarkers of tumor phenotype and aggressiveness. CEMIP, originally called KIAA1199/CCSP1, is one such gene that is highly upregulated in colon cancer [5-8]. The role of CEMIP in colon cancer progression and mediating oncogenic growth is unclear, with studies implicating CEMIP as a target of Wnt/β-catenin signaling [5, 7, 9], and as a promoter of glycogen breakdown and cellular survival [10]. Recently, a study by Yoshida *et al*. demonstrated that CEMIP can bind to the glycosaminoglycan hyaluronan (HA), and is essential for the degradation of HA by human fibroblast cells [11]. HA is a large, linear molecule (>1000 kDa) made up of repeating disaccharide units and is abundantly expressed in most tissues, including the colon [12]. Degradation of HA to low molecular weight fragments can stimulate angiogenesis, promote cell migration, and play an important role in cancer progression and inflammation [13-16], thus suggesting a possible functional role for the upregulation of CEMIP in the progression of colon cancer.

In this study, we comprehensively characterize CEMIP expression in colon cancer and investigate its role in colon cancer progression and as an indicator of poor clinical outcome. We identify CEMIP as a secreted protein that is induced as early as the colon adenoma stage, whose overexpression is associated with poor clinical outcome in colon cancer patients, and has a potential role in promoting tumor metastasis.

## RESULTS

### *CEMIP/KIAA1199* expression is induced in colon neoplasia

**Figure 1:**
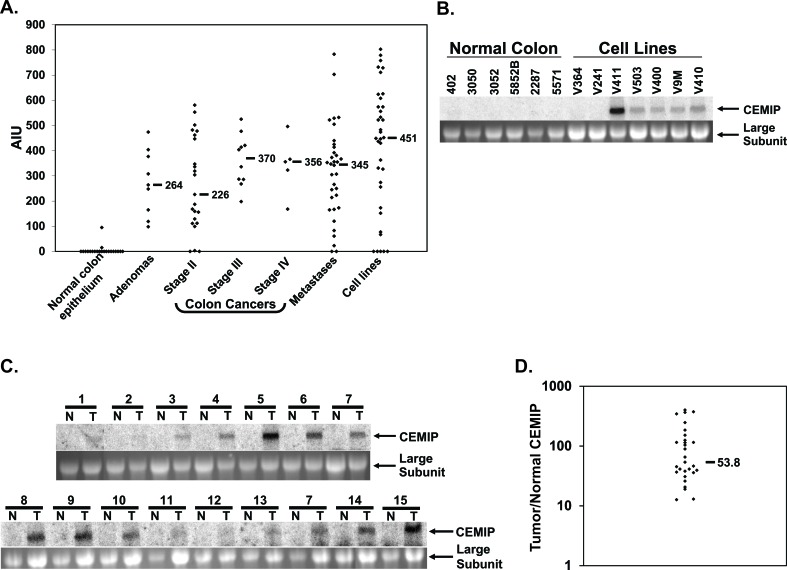
CEMIP mRNA expression in normal colon epithelium and colon cancer samples **A.** Expression levels of CEMIP measured on GeneChip microarrays for samples of normal colon epithelium, colon adenomas, colon cancer primary tumors of stages II, III and IV, colon cancer hepatic metastases, and colon cancer cell lines. Horizontal bars denote median expression values within each group. Transcript hybridization to expression microarrays is measured in Average Intensity units (AIU). **B.** Northern blot analysis of CEMIP expression in 6 normal colon epithelium samples *versus* colon cancer cell lines (upper panel). **C.** Northern blot analysis of CEMIP expression in 15 samples of colon cancer tissue (T) and paired normal colonic mucosa (N), upper panels. The lower panels are the ethidium bromide stains of the 28S ribosomal RNA subunit for each of the corresponding samples. **D.** Real-time PCR measurement of CEMIP transcript expression. Shown is the ratio of CEMIP expression in colon cancer *versus* matched normal colon mucosa for 29 patients. CEMIP values are normalized against expression of the house-keeping gene Beta-2-microglobulin. Horizontal black bar denotes mean value.

To identify novel markers of colon neoplasia, we used GeneChip gene expression microarrays to compare genomewide patterns of gene expression in colon tumors *versus* normal colon epithelium[17]. Twenty-one normal colonic mucosal samples were compared to 72 primary colon tumors and 36 colon cancer cell lines on DNA microarrays [17]. The two most highly induced probesets corresponded to P-Cadherin, already known to be induced in colon cancers [18], and to *KIAA1199* (herein called *CEMIP*), which was a hypothetical gene with only a partial cDNA at the time these expression array studies were performed. As shown in Figure 1, while only two normal colon mucosal samples showed any expression of CEMIP above the microarray measurement threshold of 25, median expression of CEMIP reached 451 in colon cancer cell lines, and 330 in primary colon tumors (Figure 1A). High levels of CEMIP were detected in early node-negative stage II colon cancers (median value 226), and in colon adenomas (1 tubular, 2 tubulovillous and 6 villous all > 1 cm in size, median value 264) (Figure 1A).

At the time of our initial studies, KIAA1199/CEMIP was reported as a 5kb partial cDNA containing a putative stop codon, but no start codon, that mapped to chromosome 15q [19]. Using, RT-PCR we connected CEMIP to additional multiple ESTs that mapped to the 15q24-25 genomic region, finding expression in colon cancers of a 4083 bp full-length coding transcript that covers 30 exons and encodes a protein of 1361 amino acids (Supplementary Figure S1A & S1C). We identified 7.0 and 7.2 kb forms of the transcript, arising from alternate splicing difference in the 5′ UTR (Supplementary Figure S1A & S1B). Both transcripts have an in-frame TAG (7.0 kb form) or TGA (7.2 kb form) stop codon 5′ to the same ATG start codon. We deposited the sequences encoding these transcripts in 2004 as GenBank accession numbers AY581148, AY585237, and AY581149, under the name Colon Cancer Secreted Protein 1 (*CCSP1*) before the gene name was officially changed to *CEMIP*.

Blast analysis of the predicted CEMIP encoded protein revealed a 42% amino acid identity to human transmembrane protein-2 (TMEM2), a widely expressed protein of unknown molecular function [20]. CEMIP homologues were also identified in the mouse, at 91% amino acid identity, and in the rat, at 90% amino acid identity.

### CEMIP expression is commonly induced in colon cancer tissues and cell lines

Northern analysis strongly confirmed that CEMIP is expressed by malignant but not normal colon tissues (Figure 1B), with a single 7 kb CEMIP transcript of moderate to strong intensity detected in 5 of 7 colon cancer cell lines, but in none of 6 normal colon epithelial tissue samples. Both cell lines negative for CEMIP expression by Northern analysis were also negative on microarrays. In further analysis of primary colorectal tumors, 13 of 15 colon cancers demonstrated easily detectable CEMIP expression; whereas, no signal was detected in any of the 15 matched normal colon mucosa samples, (Figure 1C). Real-time PCR analysis of a second independent set of 29 colon cancer cases further confirmed CEMIP induction in colon cancers. All 29 cancers showed a greater than 12-fold increase in CEMIP expression over matched normal colon mucosa, with a median increase of 54-fold (Figure 1D).

### Detection of endogenously expressed CEMIP protein

**Figure 2:**
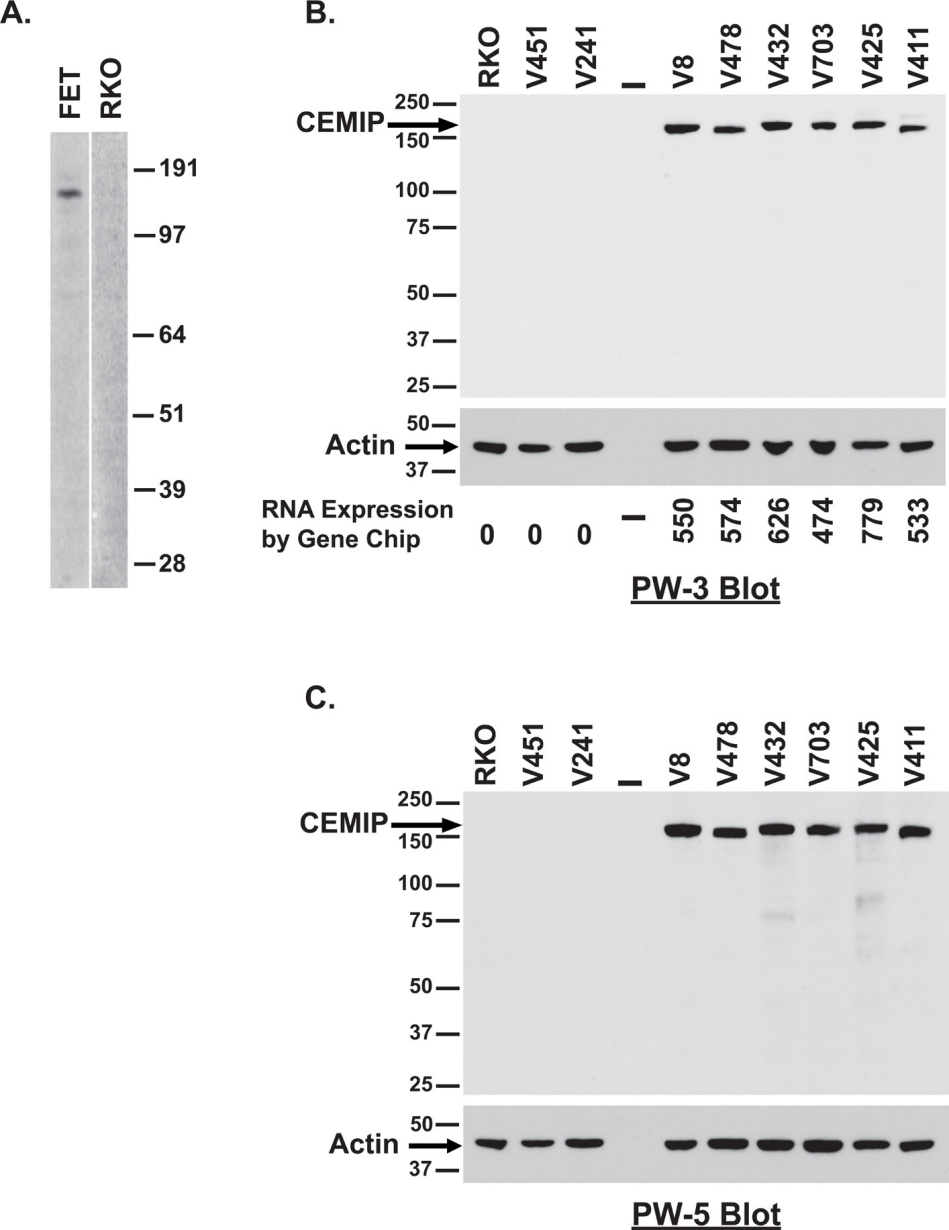
Detection of endogenous CEMIP protein in colon cancer cell lines **A.** Western blot analysis of lysates from CEMIP transcript expressing FET colon cancer cells *versus* CEMIP non-expressing RKO colon cancer cells using anti-CEMIP monoclonal antibody PW-3. **B.**-**C.** Western blot analysis of lysates for CEMIP transcript expressing lines *versus* non-expressing lines using anti-CEMIP monoclonal antibody PW-3 **B.** or anti-CEMIP monoclonal antibody PW-5 **C.**. Blotting for actin was used to control for sample loading. Corresponding mRNA expression levels are indicated below panel 2B.

To interrogate induction of a *CEMIP* encoded protein product, we purified recombinantly made CEMIP protein and developed anti-CEMIP monoclonal antibodies. Specificity of our lead monoclonal antibody, designated PW-3, was confirmed by its detecting only the correctly sized ∼153kD CEMIP protein band in Western blots of FET colon cancer cells that are positive for CEMIP transcript, *versus* detecting no protein bands in RKO colon cancer cells that are CEMIP transcript negative (Figure 2A). CEMIP overexpression at the protein level in colon cancer cell lines was confirmed by Western blot with the detection of CEMIP in an additional 6 colon cancer cell lines that were positive for mRNA overexpression and no detection of CEMIP in 2 colon cancer cell lines negative for CEMIP mRNA expression (Figure 2B). Specificity of the PW-3 antibody for detecting CEMIP was further established in an independent Western blot of the same samples using an independently developed monoclonal antibody, PW-5 (Figure 2C). Additionally, deletion of *CEMIP* in a CEMIP expressing cell line resulted in no CEMIP protein being detected by Western analysis with PW-3 antibody (Figure 6C).

**Figure 3:**
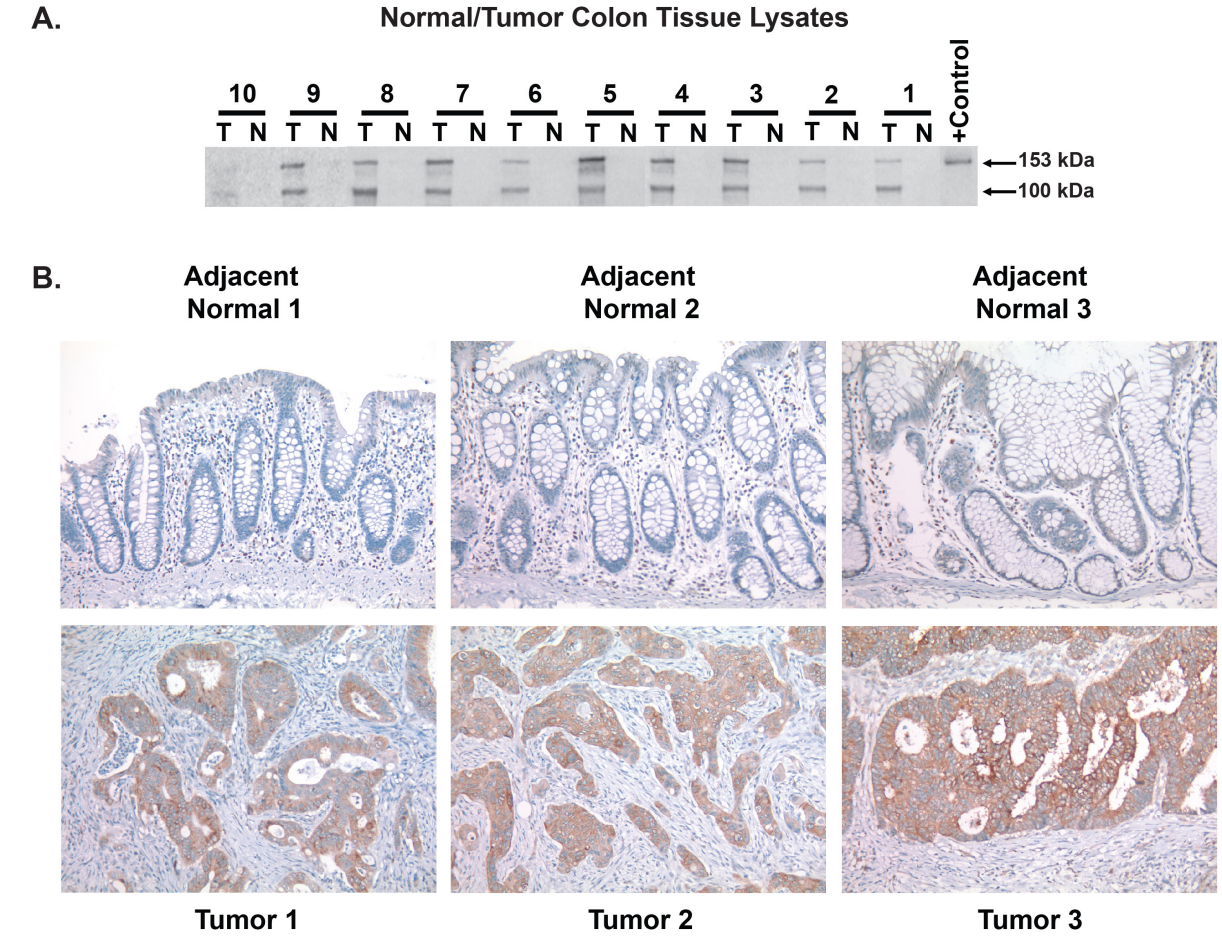
Induction of CEMIP protein in patient colon tumor samples **A.** Detection of endogenous CEMIP protein by serial immunoprecipitation and Western blot analysis using monoclonal antibody PW-3 on lysates from colon cancer tumor tissues (T) *versus* matched normal colonic mucosa (N)from 10 different colon cancer patients. Purified T7 epitope tagged CEMIP protein serves as a positive (+) control. **B.** Immunostaining of CEMIP protein using anti-CEMIP monoclonal antibody, PW-3, in 3 cases of colon cancer tumors *versus* adjacent normal colonic mucosa.

Having confirmed induction of CEMIP protein expression in colon cancer cell lines, we next determined if CEMIP protein levels are upregulated in patient colon tumors. Serial CEMIP immunoprecipitation and Western blot analyses from 10 cases confirmed that, identical to the CEMIP transcript, endogenous CEMIP protein is absent in normal colonic mucosa but is strongly induced in colon cancer tumors (Figure 3A). In colon cancer tumors, we detected CEMIP protein at the expected ∼153kD molecular weight along with a second 100kD molecular weight species. In colon cancer cell lines, only the 153kD species was detected (Figure 2A-2B), suggesting that the 100kD species is either a proteolytic fragment of the mature 150kD CEMIP molecule, or represents a yet unknown CEMIP splice variant.

The marked induction of CEMIP protein in colon cancer tumors was further confirmed by immunohistochemistry, which strongly detected CEMIP protein in colon cancer cells in multiple colon cancer tumors tested, and showed absence of detectable CEMIP in each matched normal colonic mucosa (Figure 3B). The specificity of the PW-3 monoclonal antibody for immunostaining was confirmed by PW-3 showing strong reactivity on immunostaining pellets of CEMIP transcript positive FET colon cancer cells *versus* no staining of CEMIP transcript negative RKO colon cancer cells (Supplementary Figure S2).

### CEMIP encodes a secreted protein

**Figure 4:**
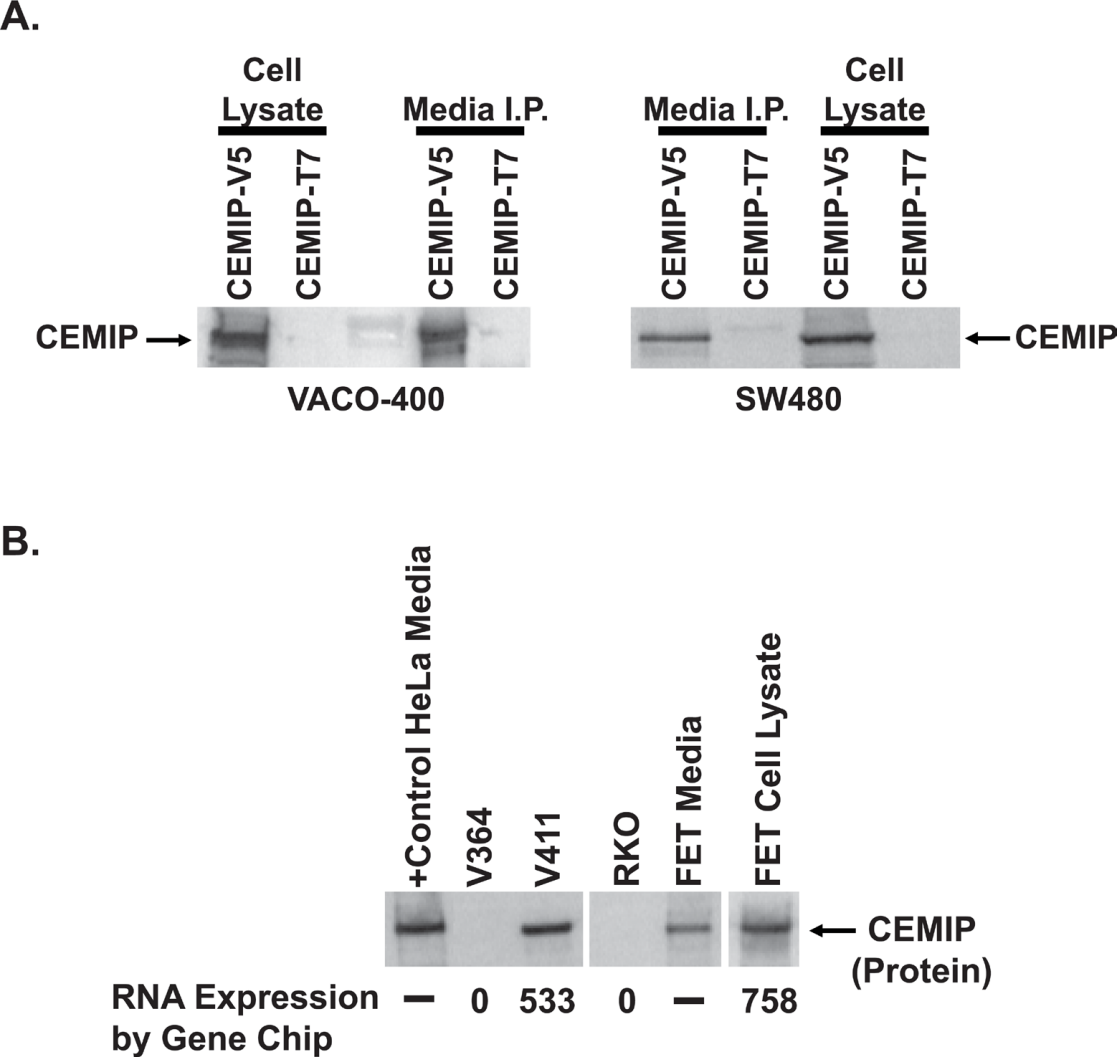
Secretion of CEMIP protein **A.** Western blot assay of CEMIP protein in lysates of CEMIP transfected cells (Cell Lysate) *versus* in the immunoprecipitates from a corresponding amount of cell culture media (Media I.P.). SW480 and VACO-400 cells were transfected with expression vectors encoding either V5 epitope tagged CEMIP (CEMIP-V5) or T7 epitope tagged CEMIP (CEMIP-T7). Immunoprecipitation and Western blotting were performed using antibodies against the V5 epitope-tag, with CEMIP-T7 samples serving as a negative control. An arrowhead denotes the position of the ∼150kDa CEMIP protein detected in both cell culture medium and lysates of CEMIP transfected cells. **B.** Detection of endogenous CEMIP protein secreted from colon cancer cells using serial immunoprecipitation and Western blot analysis. Shown are assays of CEMIP protein from 1 ml of cell culture media from colon cancer cell lines FET and V411 that express CEMIP transcript, *versus* from cell lines V364 and RKO that are negative for CEMIP transcript expression. Corresponding RNA expression levels are indicated below the panel. Also shown is an assay of cell culture media and matched cell pellet lysate from CEMIP expressing FET cells. Samples of FET cells assayed represent 3% of the total FET cell pellet and 2% of the corresponding FET media. Media from CEMIP transfected HeLa cells, which do not endogenously express CEMIP, serves as a positive control.

Analysis of the predicted CEMIP protein sequence employing InterProScan [21] identified one G8 domain and two GG domains, both with no known function [22, 23]. However, analysis using the SignalP version 3.0 [24], PSORT II algorithms [25], TMHMM [26], and TMMOD [27], identified a putative N-terminal signal peptide sequence, comprising the first 30 to 34 amino acids of CEMIP, but no predicted transmembrane domain, suggesting that CEMIP may be a secreted protein. To test this prediction, we transfected VACO-400 and SW480 colon cancer cell lines with expression vectors encoding C-terminal V5 or T7-epitope-tagged CEMIP. Antibodies against the V5 epitope tag detected a CEMIP protein with a molecular weight of ∼153kDa in the clarified cell culture media supernates collected from both cell lines, with 50% of the tagged CEMIP protein detected in the media and the remaining 50% of tagged CEMIP protein segregating with the pelleted transfected cells (Figure 4A). Cells transfected with an expression vector for a T7-epitope-tagged CEMIP protein served as a negative control for assays directed against the V5-tagged CEMIP (Figure 4A).

To further examine CEMIP protein secretion, we next tested the ability of anti-CEMIP monoclonal antibodies to immunoprecipitate endogenously expressed full-length CEMIP protein. Consistent with our findings in the tagged CEMIP transfection studies, anti-CEMIP monoclonal antibodies immunoprecipitated endogenous full length CEMIP protein from the serum-free cell culture supernatant of colon cancer cell lines expressing CEMIP (FET and V411) while no CEMIP was detected in lines that do not express the CEMIP transcript (RKO and V364) (Figure 4B).

### Colon cancers that overexpress CEMIP show markedly reduced survival

**Figure 5:**
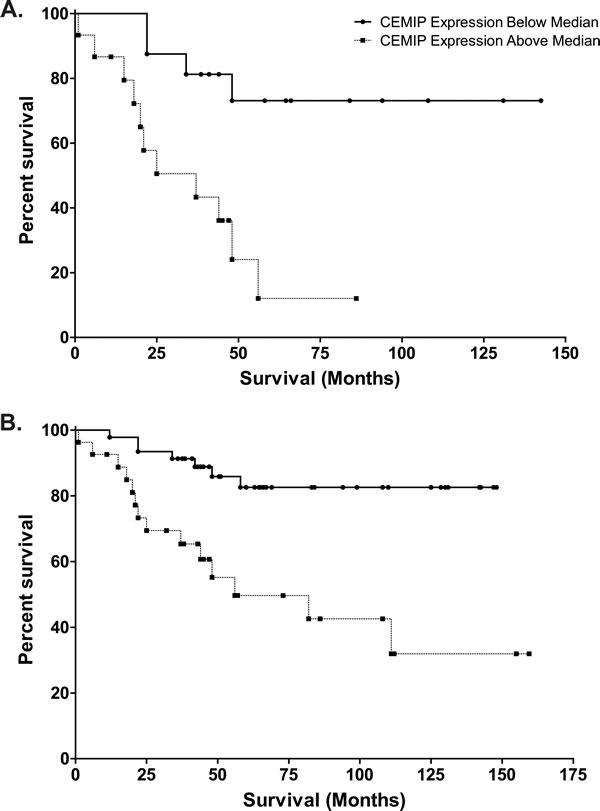
Kaplan-Meier analyses of survival in CEMIP high (values greater than 1.024, selected as median of stage III colon cancer cases) *versus* CEMIP low (values less than 1.024) colon cancer cases **A.** Survival curve of 31 stage III colon cancer patients with tumor CEMIP expression levels above (dashed line, *n* = 16) or below (solid line, *n* = 15) the median value of 1.024, demonstrating decreased survival in those with high tumor CEMIP transcript levels (*P* = 0.004). **B.** Survival curve of 73 stage II and stage III colon cancer patients with tumor CEMIP expression levels above (dashed line) or below (solid line) 1.024, demonstrating decreased survival in those with high tumor CEMIP transcript levels (*P* = 0.0003).

Since CEMIP expression is highly upregulated in colon cancer, we examined if CEMIP expression levels might be prognostic of a patient's clinical outcome. CEMIP mRNA expression was measured by real-time PCR of colon cancer tumors obtained from 31 stage III colon cancer patients with microsatellite stable cancers and for whom long term clinical follow-up was available. Colon cancer cases were divided into those with CEMIP expression above the median (CEMIP high), and those with CEMIP expression below the median (CEMIP low) (median CEMIP = 1.024). Kaplan-Meier survival analysis for colon cancer specific death showed that CEMIP low cases (*n* = 15) had notably favorable outcomes, with median survival of greater than 140 months. In contrast, CEMIP high cases (*n* = 16) demonstrated markedly worse outcomes, with median survival of only 37 months, a reduction of 8.6 years (*P* = 0.004) (Figure 5A). Multivariable Cox regression survival modeling adjusting for age at diagnosis, gender, and race showed that those with CEMIP expression values greater than or equal to the median had 4.93 fold increased risk of death as compared to those with values below the median (HR = 4.93, 95% CI = (1.50,16.14)). Kaplan-Meier and multivariable Cox regression survival models for all deaths showed similar results (data not shown).

The adverse outcome associated with high CEMIP tumor expression was also evident in an analysis that combined the 31 stage III colon cancer cases with an additional 42 stage II colon cancer cases that were also microsatellite stable and had available long term follow-up. In this analysis, we retained the same definition of CEMIP high *versus* low cancers as in our original analysis (i.e. the new colon cancer cases were defined as CEMIP high or low using the same criteria of having CEMIP expression higher or lower than 1.024). In Kaplan-Meier survival analysis of this combined cohort of 73 colon cancers cases, patients with CEMIP low expression again showed favorable outcomes, with median survival time greater than 148 months; whereas, cases with CEMIP high expression, again, showed a much reduced median survival time of 56 months (Figure 5B). Moreover, this combined cohort showed a 10-fold increase in the level of statistical significance for the differences in outcome between CEMIP high *versus* low groups (*P* = 0.0003 for CEMIP effect on survival in stage II plus III cases *versus P* = 0.004 for stage III cases only) (Figure 5B). While the small number of events in the stage II cohort precludes meaningful statistical analysis in stage II only, the increased significance for the survival difference in the combined stage II plus stage III group provides added support for high CEMIP expression being associated with adverse outcome.

### Deletion of CEMIP inhibits colon tumor growth in a mouse xenograft model

**Figure 6:**
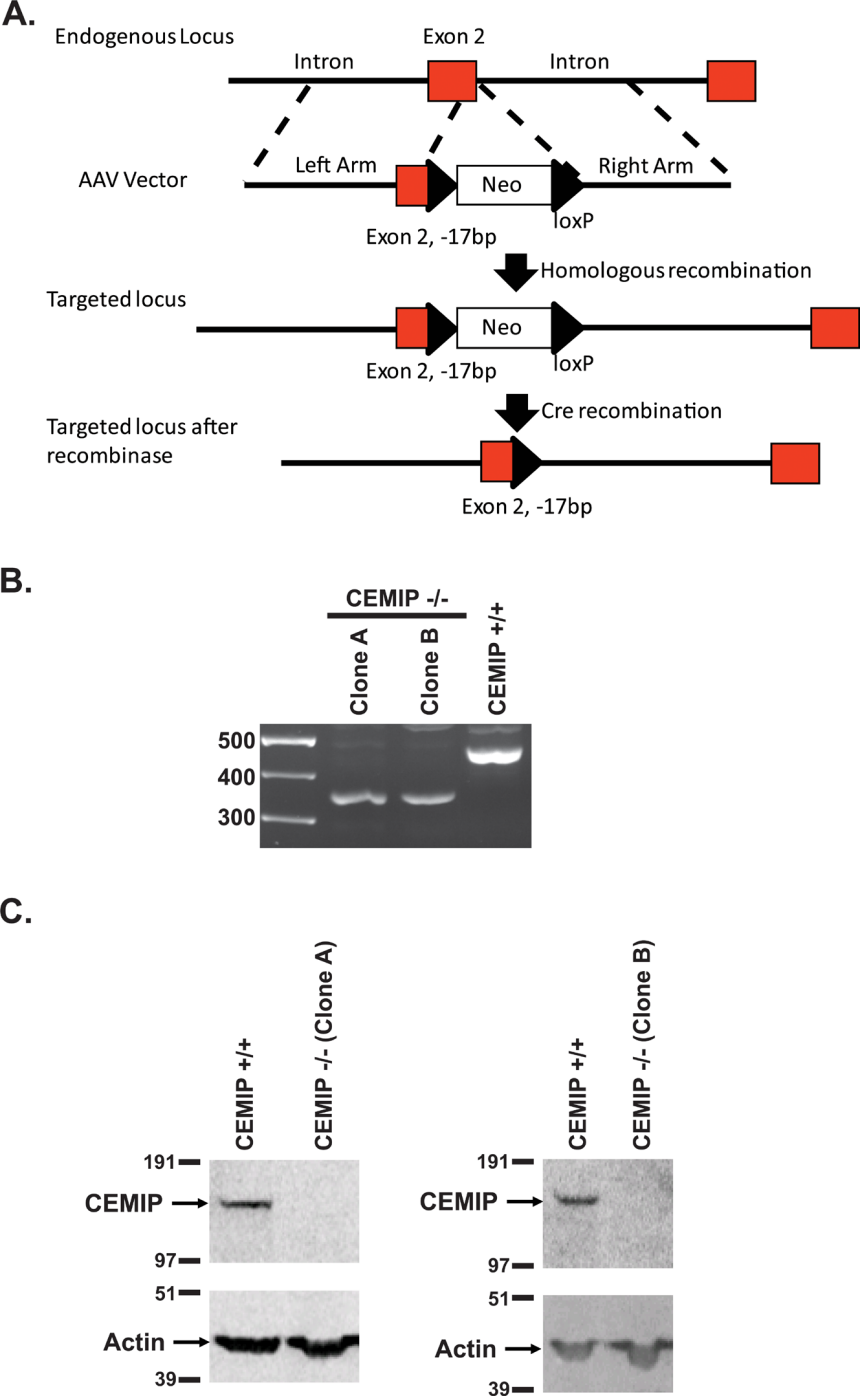
Gene knockout of CEMIP in DLD-1 cells **A.** Schematic diagram for targeting exon 2 for deletion in *CEMIP*. **B.** RT-PCR confirmation for deletion of exon 2 in *CEMIP* deleted DLD-1 clones (Clone A and Clone B). The PCR primers span exon 2 and the expected band size for exon 2 deleted cells is 343bp *versus* 453bp for non-targeted DLD-1 cells (*CEMIP* +/+). **C.** Western blot for CEMIP protein in deleted clones A and B showing a lack of a 150 kDa band, whereas a band is detected in non-targeted DLD-1 cells (CEMIP +/+). Blotting for actin was used to control for sample loading.

**Figure 7:**
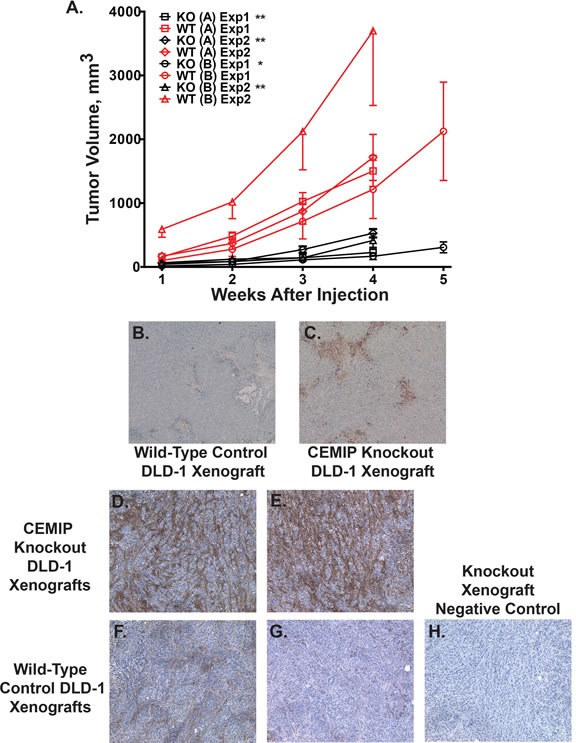
Reduced tumor growth and increased apoptosis in CEMIP negative tumor xenografts **A.** Xenograft growth curves in athymic mice injected with CEMIP knockout DLD-1 cells (black lines) or wild-type DLD-1 cells (red lines). Two separate knockout clone lines were derived and xenograft growth for each line was tested in two separate experiments. The symbols for respective experiments are as follows, (square) CEMIP knockout clone A experiment 1, (diamond) knockout clone A experiment 2, (circle) CEMIP knockout clone B experiment 1, (triangle) knockout clone B experiment 2. Matched wild-type DLD-1 xenograft controls for each experiment have the corresponding symbol, but in red. Error bars are standard errors of the mean. (*) Denotes *P* < 0.05 for knockout *versus* matched control for all time points while (**) denotes *P* < 0.01 for all time points. **B.**-**C.** Shown is immunostaining for cleaved caspase-3 in harvested xenografts from mice injected with wild-type, CEMIP expressing DLD-1 cells **B.**, or CEMIP knockout DLD-1 cells **C.**. **D.**-**H.** Shown is histochemical staining for HA in harvested xenografts from mice injected with CEMIP knockout DLD-1 cells **D.-E.**, or CEMIP expressing DLD-1 cells **F.**-**G. H.** HA negative control stain of CEMIP knockout DLD-1 cells in which the biotinylated HA binding protein is omitted.

CEMIP did not demonstrate focus forming activity in NIH3T3 cells (by transfection), or change in anchorage independent growth (by adding CEMIP protein), and attempts to express CEMIP protein by transfection in those rare colon cancer cell lines that did not induce CEMIP were in general unsuccessful. To interrogate the contribution of CEMIP to colon cancer phenotype, we used a gene targeting vector [28] to knock out *CEMIP* in the colon cancer cell line, DLD-1, that normally expresses CEMIP at high levels. A 17 bp deletion was introduced into both copies of *CEMIP* exon 2 that contains the start ATG and signal peptide sequence. This 17 bp deletion of exon 2 (plus 2 bp of the immediately following intron) results in only the first 25 amino acids of CEMIP being properly translated, with the remainder (1336 aa) being out of frame (Figure 6A). Two independent DLD-1 clones were obtained in which both alleles of *CEMIP* were knocked out as determined by genotyping assays, and in which no CEMIP protein was detected by Western analysis (Figure 6B and 6C). On plastic, CEMIP deleted clones showed slightly slower growth rates than wild-type cells, with numbers of CEMIP deleted cells being approximately 45% that of wild-type DLD-1 at 7 days after plating (Supplementary Figure S3). The effect of deleting CEMIP was, however, markedly amplified when the two CEMIP null clones were injected subcutaneously into athymic nude mice. As demonstrated in Figure 7A, tumors from both CEMIP knockout clones grew significantly slower in mice than did tumors from wild-type CEMIP positive cells, with these findings replicated in duplicate experiments for each clone (Figure 7A, *P* < 0.01 for all time points except for clone B experiment 1, which was *P* < 0.05 for all time points). To investigate the cause of decreased tumor growth in CEMIP negative cells *in vivo*, we examined CEMIP expressing and CEMIP negative tumors by immunostaining for markers of apoptosis (cleaved caspase-3), proliferation (Ki-67), leukocyte infiltration (CD45), and vascularization (CD31). A marked increase in cleaved caspase-3 was detected in tumors from CEMIP negative DLD-1 cells, suggesting that knocking out CEMIP impedes tumor growth by inducing increased apoptosis (Figure 7B-7C). No differences were detected in Ki-67, CD45 or CD31 immunostaining between CEMIP expressing *versus* CEMIP negative tumors (data not shown).

### Knocking out CEMIP increases tumor hyaluronan (HA)

A recent study by Yoshida *et al*. suggests that CEMIP plays a role in degrading HA, a glycosaminoglycan that is one of the major components of the extracellular matrix [11]. Because remodeling of the extracellular matrix is crucial for aspects of tumor biology including tumor expansion, neovascularization, invasion, and metastasis [29], we tested if CEMIP mediated this function in colon cancers by histochemical staining for HA in the aggressive xenografts formed by CEMIP wild-type DLD1 colon cancer cells *versus* the impeded xenografts formed by CEMIP knockout cells. As shown in Figure 7D-7H, a marked increase in HA levels was detected in the impeded tumors from CEMIP negative DLD-1 cells (Figure 7D-7E) as compared to tumors from CEMIP expressing DLD-1 cells (Figure 7F-7G). Thus CEMIP overexpression is key mediator of the ability of colon cancer cells to degrade the HA component of extracellular matrix.

## DISCUSSION

In the present study, we have comprehensively characterized CEMIP expression in colon cancer and have provided the novel findings that CEMIP overexpression is associated with poor patient prognosis and is necessary for tumor growth. We originally identified KIAA1199 as a novel transcript highly induced in colon cancer and deposited the full-length transcript and 5′ UTR variants as GenBank accession numbers AY581148, AY585237, and AY581149, under the name Colon Cancer Secreted Protein 1 (*CCSP1*) before the gene name was officially changed to *CEMIP*. We found that CEMIP is markedly upregulated in colon cancer both at the mRNA and protein level, with induction occurring as early as the colon adenoma stage, and encodes a secreted protein (U.S. patent #7,118,912) [8]. Similar to ours and others [5-7] findings in colon cancer, a search of the Oncomine [30] database identifies microarray studies demonstrating upregulation of CEMIP in several other epithelial cancers, including breast [31], gastric [32], and pancreatic cancer [33]. These data suggest that induction of CEMIP expression in neoplasia may be a general feature of many solid tumors. We found no evidence of amplification of *CEMIP* or for rearrangement of the gene promoter as possible explanations for CEMIP overexpression in colon cancer. Similarly, CEMIP was not found to be a target for amplification in the TCGA dataset [34]. Findings by Sabates-Bellver *et al*., suggests that CEMIP overexpression in colon tumors may be due to activation of the Wnt signaling pathway, consequent to mutations in either APC or beta-catenin [7]. This observation is consistent with our findings that induction of CEMIP is common in colon cancers and occurs as early as the colon adenoma stage.

Important to this study is our finding that elevated CEMIP expression in human colon tumors is associated with markedly reduced survival in stage III colon cancer cases. Individuals whose stage III colon cancer tumors express CEMIP below the median lived an average > 8.6 years longer than stage III colon cancer cases whose tumor CEMIP levels were above the median, a dramatic difference in patient survival. Multivariable Cox regression analysis including age at diagnosis, gender, and race demonstrated that CEMIP expression was an independent prognostic factor for overall survival. Elevated tumor CEMIP expression showed an even more statistically significant association with reduced survival in an expanded set of individuals having either stage II or stage III colon cancers. Our finding of high tumor CEMIP expression associated with poor prognosis in colon cancer is supported by findings of a 50% decrease in 5-year survival of gastric cancer patients whose tumors expressed high *versus* low CEMIP [35] as well as a survey of breast cancer DNA microarray datasets finding reduced survival in patients with tumors expressing high *versus* low CEMIP [36]. Similarly, the association of CEMIP expression with poor prognosis in colon cancer was found in a different context by Birkenkamp-Demtroder *et al*. based on immunohistochemical studies in which a slightly better prognosis was reported for subgroup of stage II patients with strong nuclear, yet weak cytoplasmic, staining for CEMIP compared to cases with strong cytoplasmic, or nuclear and cytoplasmic staining [5]. Our findings of a marked difference in survival between individuals whose colon tumors have high *versus* low CEMIP in our patient cohort will clearly merit testing in follow-up studies of larger cohorts of stage III and of stage II plus III colon cancer cases, as well as in cohorts of sufficient size to detect possible effects of CEMIP expression on outcome of stage II only colon cancer cases.

Our finding that knocking out CEMIP markedly attenuates xenograft growth provides the first *in vivo* data demonstrating a critical role for CEMIP expression in colon cancer tumor growth, thus suggesting that CEMIP directly contributes to tumor phenotype and may itself be a therapeutic target. In testing for CEMIP somatic mutations, we identified only one mutation among 13 colon cancer cell lines, a homozygous Gly1173Asp alteration present in a single sample. Likewise, TCGA data finds CEMIP mutated in only 8 of 212 cases, with none of the mutations predicted to have a significant deleterious impact on function [34]. Thus, the contribution of CEMIP to tumor phenotype appears to generally be mediated by the native protein. Facilitation of tumor growth by CEMIP overexpression may be due, in part, by protecting cells from apoptosis; as deletion of CEMIP resulted in increased cleaved caspase-3 staining in CEMIP knockout DLD-1 xenografts with significantly attenuated growth. Our findings are supported by studies in other cancers demonstrating CEMIP can protect cervical [37], gastric [10], and breast [38] cancer cells from apoptosis, though the exact molecular mechanism of CEMIP's anti-apoptotic effect has yet to be elucidated. Additionally, our demonstration that CEMIP knockout results in markedly increased intratumoral levels of HA suggests an important role for CEMIP overexpression in the degradation of HA in the extracellular matrix. These findings are consistent with the biochemical studies of Yoshida *et al*. demonstrating CEMIP as a hyaladherin involved in HA depolymerization [11]. High molecular weight HA plays an important role in a variety of functions involved in maintaining tissue integrity and homeostasis while low molecular weight fragments from the degradation of HA are potent promoters of a variety of functions important to tumor progression, and have been detected in a variety of cancer types [13-16]. Of note, *in vitro* studies have demonstrated that addition of HA tetrasaccharides to cultured cell lines can induce heat shock proteins and suppress apoptosis [39], potentially linking CEMIP's anti-apoptotic activity with its role in HA depolymerization. Our finding of an inverse association between CEMIP levels and HA deposition suggests a model in which CEMIP overexpression contributes to colon cancer phenotype both by removing a HA physical barrier as well as by increasing production of tumor promoting low molecular weight HA fragments. Furthermore, it suggests that CEMIP overexpression may be a novel target for therapy as studies in which tumor HA turnover is disrupted demonstrate tumor growth inhibition both *in vitro* and *in vivo* [13, 40]

In conclusion, our findings that CEMIP is not only a prognostic marker of outcome in colon cancer, but also directly contributes to maintenance of tumor phenotype, should spur further investigations to determine the potential of CEMIP as a therapeutic target, and to elucidate its biological function in HA metabolism when overexpressed in human cancers. Future studies are currently planned to elucidate the mechanism of CEMIP expression on protection from apoptosis. Finally, the finding that CEMIP is a secreted protein whose expression is dramatically upregulated in colon adenomas and early colon cancers nominates this protein as a highly interesting candidate serological marker of early human colon neoplasia, for which future studies will certainly be warranted.

## MATERIALS AND METHODS

### Sequences

Human CEMIP mRNA and gene sequence GenBank accession numbers as deposited by our group under the name Colon Cancer Secreted Protein 1 (*CCSP1*) are AY581148, AY585237, and AY581149.

### Cell lines

VACO cell lines were established in the investigators laboratories according to previously described methods [41]. The lines are authenticated by DNA fingerprinting against original patient tumors and tested for mycoplasma contamination on an annual basis. SW480 and DLD-1 cell lines were obtained from ATCC (Manassas, VA) and the cell lines were used for experiments with minimal passages after resuscitation. All colon cancer cell lines were maintained in MEM2+ medium as previously described [42] except for DLD-1 which was maintained in McCoys medium with 10% FBS. The tetracycline-inducible HeLa cell line, T-REx™-HeLa was obtained from Invitrogen (Carlsbad, CA) and used for experiments immediately upon receiving the line. FET was a generous gift from Dr. Michael Brattain and was grown and authenticated as previously described [43].

### Human samples

The human samples were accrued under the protocol, “CWRU 7296”, and was approved by the University Hospitals Case Medical Center Institutional Review Board for Human Investigation with the assigned UH IRB number 03-94-105. Under this protocol, human samples were obtained through written informed consent from patients for research use.

### DNA expression microarray analysis

Custom expression microarrays [17, 44] and Affymetrix Human Exon 1.0 ST Arrays [45, 46] were utilized as previously described (GEO Accession: GSE1476).

### Northern blot analysis

The probe for exons 1-9 of CEMIP was amplified by PCR using the primers 5′-AGGCGTGACACTGTCTCGGCTACAG-3′ (forward) and 5′-CCACTCCACGTCTTGAACCCAC-3′ (reverse) and the analysis was performed as previously described [47].

### CEMIP real-time PCR of matched tumor and normal tissues

RNA from all tissue samples used was prepared by extraction with guanidine isothiocyanate as previously described [48]. RNA concentrations were determined using a ND-1000 Spectrophotometer (NanoDrop, Wilmington, DE) and all samples used had an A_260/280_ ratio value greater than 1.70. All reverse transcription quantitative real-time PCR assays were performed following the MIQE guidelines [49]. cDNA was synthesized from 1 μg of input RNA using AMV Reverse Transcriptase (Roche, Indianapolis, IN) following the manufactures recommended protocol and used for subsequent qPCR assays.

Real-time PCR measurement of CEMIP from paired normal and tumors samples was performed using the human hydrolysis probe/primer set Hs00378520_m1 (KIAA1199/CEMIP, NM_018689) from Applied Biosystems (Foster City, CA). A 20ul reaction mix contained 1 μl of cDNA template and a 1:20 dilution of primer/probe in 1X IQ-Supermix (Bio-Rad, Hercules, CA) and the cycling conditions were 95°C for 4 min, followed by 50 cycles of 95°C for 15 sec and 60°C for 1 min. Beta*-2-*microglobulin (B2M) was used as the reference gene for normalization and was amplified using the human B2M (NM_004048) hydrolysis probe/primer set Hs99999907_m1 from Applied Biosystems following the same reaction conditions above. The level of CEMIP expression was determined as the ratio of CEMIP:B2M = 2exp- (Cq_CEMIP_ – Cq_B2M_). For each reverse transcription reaction, Cq_CEMIP_ and Cq_B2M_ values were determined as the average values obtained from three independent real-time PCR reactions. RNA that had not undergone the reverse transcriptase step, as well as a water sample that was carried through the reverse transcriptase step, were used as negative controls. Both controls were negative for all assays performed. PCR efficiency, R^2^, slope, and *y* intercept for the calibration curve for each assay was as follows CEMIP (98.7, 0.992, −3.35, 23.01) and B2M (93.2, 0.995, −3.49, 19.07). Products from representative CEMIP PCR reactions were sequenced to confirm that the reactions actually amplified authentic CEMIP derived DNAs.

### Analysis of CEMIP expression level in stage II and stage III colon cancer cases

Identification of a reference gene set for normalizing real-time PCR of human stage II and stage III colon cancer samples are detailed in the supplementary information. Real-time PCR measurement of CEMIP from 42 stage II and 31 stage III tumor samples was performed using the human hydrolysis probe/primer set Hs00378530_m1 (KIAA1199/CEMIP, NM_018689) from Applied Biosystems (Foster City, CA) and followed the same reaction conditions as specified above. Also as above, (Cq_GEO3_), the geometric mean of the Cq values for CPNE2, SAC3D1 and TMEM160 was used for normalization. The level of CEMIP expression was determined as the ratio of CEMIP:GEO3 = 2exp- (Cq_CEMIP_ – Cq_GEO3_). For each reverse transcription reaction, Cq_CEMIP_, Cq_CPNE2_, Cq_SAC3D1_, and Cq_TMEM160_, values were determined as the average values obtained from three independent real-time PCR reactions. RNA that had not undergone the reverse transcriptase step, as well as a water sample that was carried through the reverse transcriptase step, were used as negative controls. Both controls were negative for all assays performed. PCR efficiency, R^2^, slope, and *y* intercept for the calibration curve for each normalization gene assay was as follows CPNE2 (95.8, 0.996, −3.43, 26.45), SAC3D1 (96.1, 0.996, −3.42, 28.79), and TMEM160 (100.5, 0.979, −3.31, 28.81).

### Transfection and detection of CEMIP from cell lysates and cell media

Construction of CEMIP expression vector transfected cells and detection of T7- or V5/His-tagged CEMIP are detailed in supplementary information.

### Generation of anti-CEMIP monoclonal antibodies

Recombinant CEMIP protein (see supplementary information) was used to generate anti-CEMIP monoclonal antibodies using contract services of Celliance Corporation (Norcross, GA).

### Western analysis of native CEMIP protein

Protein lysates from cell lines were prepared in RIPA buffer (1x PBS, 1% Igepal CA-630, 0.5% sodium deoxycholate, 0.1% SDS) containing Complete Mini protease inhibitor cocktail (Roche, Indianapolis, IN), were separated on a 4-12% Bis-Tris SDS-PAGE gel (Invitrogen, Carlsbad, CA) (50 μg per lane), and transferred onto Immobilon™-P PVDF membranes (Millipore, Billerica, MA). Membranes were blocked with 5% nonfat milk, probed with a 1:1200 dilution of PW-3 or 1:3000 dilution of PW-5 for the detection of native CEMIP, and a 1:100,000 dilution of α-actin (Sigma #A5441), then developed using a 1:1500 dilution of donkey anti-mouse horseradish peroxidase (Jackson ImmunoResearch Laboratories, Inc., #715-035-150). Enhanced Chemiluminescence Plus (Amersham Biosciences, Piscataway, NJ) and a STORM 840 phosphoimager were used to detect protein bands. Immunoprecipitation and western blot analysis of native CEMIP from cell line media was the same as above except that the media was precleared with Protein G beads (Upstate Biotechnology, #16-266) at 4°C for 2 h before adding a 1:40 dilution of antibody supernatant for the overnight immunoprecipitation, and the next day Protein G beads were added to each sample and rocked at 4°C for 1.5 h. The samples were then washed 3 times with RIPA buffer before loading.

Protein lysates from frozen human tissues were obtained by pulverizing a sample in a chilled metal tissue pulverizer and scraping the powder into chilled Pierce T-PER^®^ lysis buffer (Pierce, Rockford, IL) containing both protease and phosphatase (Sigma, St. Louis, MO) inhibitors. The samples were then incubated for 20 min at 4°C and were pipetted several times to ensure complete lysis. Finally, the samples were centrifuged for 5 min at 10,000 rpm and the clarified supernatants were aliquoted into fresh, chilled tubes and then stored at −80°C. The immunoprecipitation/western blot analysis was the same as for the detection of CEMIP from cell line media, except that 1.0 mg of protein was used for the colon normal and tumor samples.

### CEMIP immunohistochemistry

Five μM-thick formalin-fixed paraffin-embedded tissue sections were baked at 60°C for 75 min, deparaffinized, and rehydrated. Antigen retrieval was performed by steaming at 98.5°C for 5 min in 10 mM citrate buffer (pH 6.0), plus a cool-down period of 20 min. Reduction of peroxidases was accomplished by incubating in 3% H_2_O_2_ in water for 30 min at room temperature. Nonspecific protein blocking (Serum-Free Protein Block, Dako, Carpenteria, CA) was performed for 60 min. Monoclonal antibodies from hybridomas that were positive for anti-CEMIP activity were purified from mouse ascites and screened to identify those reactive against CEMIP in an immunohistochemical assay. One such antibody, PW-3, was identified that stained cell pellets from FET colon cancer cells that express endogenous CEMIP, but did not stain cell pellets from a non-expressing colon cancer cell line (RKO) (Supplementary Figure S2), and that further identified only a single protein band corresponding to CEMIP on western analysis of FET cells (Figure 2B). The antibody was diluted (1:150) in 1% BSA (Roche) and incubated overnight at 4°C in humidified chambers. The slides were washed thoroughly, and Protein Block was added again for 30 min. Envision ^TM^+ HRP Anti Mouse kit (Dako, Carpenteria, CA) was used for development, applying secondary antibody conjugated to a polymer-HRP, following manufacturer's protocol. Staining was performed with diaminobenzidine (DAB)+ substrate-chromogen (Dako, Carpenteria, CA), which was added to the slides for 7 min. All washes were done with TBST (50 mM Tris·HCl, 150 mM NaCl, 0.05% Tween, pH 7.6) diluted in deionized water. The sections were then counterstained by using Harris modified hematoxylin stain (Fisher Scientific, Pittsburgh, PA) for 1 min, dried and mounted.

### Construction of CEMIP deleted DLD-1 cells

Construction of the targeting vector and procedure for knocking out CEMIP using a recombinant adeno-associated virus system were performed as described in reference [28] using the schema shown in Figure 6.

### Xenograft growth studies

Mouse studies were performed in the Case Animal Resource Center under a protocol approved by the Institutional Animal Care and Use Committee. Athymic female nude mice, 4–6 weeks of age, were injected subcutaneously on each flank with 5 × 10^6^ CEMIP negative DLD-1 cells or the control parental DLD-1 cells (*n* = 5 mice for each condition). Mice were sacrificed 4-5 weeks after injection and the tumors were isolated, formalin-fixed paraffin-embedded, and sectioned for immunostaining.

### Ki-67, CD31, CD45, and cleaved caspase-3 immunohistochemistry

The antibodies Ki-67 (Dako, #M7187), Cleaved Caspase-3 (Cell Signaling, #9661), CD31 (Abcam, #ab28364), and CD45 (R&D Systems, #MAB114) were used for immunostaining. Immunostaining was similar as described above for CEMIP and is detailed in supplementary information.

### Histochemical staining for hyaluronan (HA)

Sections were cut, deparaffinised, and rehydrated as described above. Antigen retrieval was performed for 30 s at 123°C in Antigen Unmasking Solution (Vector Laboratories, Burlingame, CA) using a pressure cooker followed by a cool-down period of 20 min. Reduction of peroxidases was accomplished by incubating for 8 min in Peroxidazed 1 (BioCare Medical, Concord, CA). Slides were sequentially blocked in Avidin-Biotin Blocking Kit (BioCare Medical), Background Sniper (BioCare Medical) and Rodent Block M (BioCare Medical) for 15 min, respectively. Biotinylated Hyaluronic Acid Binding Protein (Millipore, Billerica, MA) was added at a 1:800 dilution and incubated for 1 h at room temperature followed by a 10 min incubation with 4+ Streptavidin HRP Label (BioCare Medical). Staining was performed with Betazoid DABKit (BioCare Medical) for 5 min followed by counterstaining with CAT Hematoxylin (BioCare Medical) for 1 min.

### Statistical analysis

Differences in gene expression levels by stage of colon cancer and differences in tumor size by CEMIP expression status were assessed using t-tests with two-sided p-values. For animal studies, sample size was determined to give 80% power for detecting a significant difference of *P* < 0.05. Survival analysis by CEMIP median status was performed using Kaplan-Meier analysis generating median survival times, and differences between survival curves were tested using the log rank test. Multivariable survival analysis was performed using the Cox proportional hazards regression model generating hazard ratios (HR) with 95% confidence intervals (95% CI).

## SUPPLEMENTARY MATERIAL FIGURES AND TABLE


