# Supplementary material for: Induction of KIAA1199/CEMIP is associated with colon cancer phenotype and poor patient survival
Source: Oncotarget. 2015 Sep 30;6(31):30500–15. doi: 10.18632/oncotarget.5921 (PMC4741547; doi:10.18632/oncotarget.5921)
Supplement: Supplementary file 1 [file oncotarget-06-30500-s001.pdf]

Induction of KIAA1199/CEMIP is associated with colon cancer phenotype and poor patient survival

Supplementary Material

SUPPLEMENTARY METHODS

Identification of a reference gene set for normalizing real-time PCR of human colon cancer samples. To identify the most stable reference transcripts for normalizing real-time PCR assays of colon cancer tumors, we mined gene expression profiles from 72 colon cancer samples that were analyzed on Affymetrix Human Exon 1.0 ST arrays. Eight transcripts were selected based on having a coefficient of variation <0.30 and uniform microarray expression that was >100 average signal intensity. Stability of these eight transcripts (DDA1, SAC3D1, TMEM160, CPNE2, TMEM134, ZNF787, ZNF746, SIRT3) was further tested by real-time PCR analysis of 28 colon cancer tumors using hydrolysis probe/primer sets from Applied Biosystems following the same reaction conditions as listed in the main text. The real-time PCR results were examined using the software programs BestKeeper [1], Normfinder [2], and genorm^{PLUS} [3] to determine the top four stable transcripts from each program (Supplemental Table 1). Copine II (CPNE2), SAC3 domain-containing protein 1 (SAC3D1) and Transmembrane protein 160 (TMEM160) were selected as reference transcripts based on being in the top four most stable transcript lists for all three programs (CPNE2 and TMEM160) or for two of the three programs (SAC3D1) (Supplemental Table 1). The hydrolysis probe/primer sets for CPNE2 (NM_152727), SAC3D1 (NM_013299), and TMEM160 (NM_017854) are Hs00541611_m1, Hs01017027_m1, and Hs00215289_m1 respectively, from Applied Biosystems. Supplementary Fig. S4 shows the individual Cq values of each of these transcripts, and the geometric mean of the Cq values for all three genes (Cq_{GEO3}) as determined in amplifications from 1 µg of input RNA from each of the tumors. Note that while highly

stable across colon cancer tissue samples, these reference genes are not suitable as an internal standard for comparison of gene expression between normal colon tissues and colon cancers.

Construction of CEMIP expression vectors. Human full-length CEMIP was amplified from cDNA and cloned into the pcDNA3.1/V5-His© TOPO® TA expression vector using the primers 5′-CGTGACACTGTCTCGGCTACAGAC-3′ (forward) and 5′-CAACTTCTTCTTCTTCACCACAG-3′ (reverse) for the V5/His-tagged construct, while the full-length, T7-tagged construct was cloned into the vector using the primers 5′-CGTGACACTGTCTCGGCTACAGAC-3′ (forward) and 5′-<u>TCAACCCATTTGCTGTCCACCAGTCATGCTAGCCAT</u>CAACTTCTTCTTCTTCACCACAG-3′ (reverse), which contains an incorporated T7-tag sequence (underline) followed by a stop codon. Sequence of the tagged constructs was confirmed by sequencing both strands (Cleveland Genomics, Cleveland, OH).

Transfection and detection of CEMIP from cell lysates and cell media. SW480 and VACO-400 cells were transfected with either T7- or V5/His-tagged CEMIP expression vector using Fugene (Roche, Indianapolis, IN) for SW480 or Effectene (Qiagen, Valencia, CA) for VACO-400 following the manufacturer's protocols. Seventy-two hours after transfection, the media was removed and clarified by centrifugation for 5 min at 2,000 rpm and the supernatant was collected in a fresh tube. Epitope-tagged CEMIP was immunoprecipitated from media samples using either a 1:1000 dilution of mouse, anti-T7 antibody (Novagen, #69522-3) or a 1:333 dilution of mouse, anti-V5 antibody (Invitrogen, #46-1157) and rocking overnight at 4°C. The next day Protein G beads (Upstate Biotechnology, #16-266) were added to each sample and rocked at 4°C for 1.5 h. The samples were then washed 3 times with RIPA buffer (1x PBS, 1% Igepal CA-630, 0.5% sodium deoxycholate, 0.1% SDS) containing Complete Mini protease inhibitor cocktail (Roche, Indianapolis, IN) and loaded onto a 4-12% Bis-Tris SDS-PAGE (Invitrogen, Carlsbad, CA) for protein separation. The accompanying pellet of transfected cells were similarly lysed for 15 min at 4°C in RIPA buffer containing protease

inhibitor cocktail. Lysates were then centrifuged for 10 min at 14,000 rpm at 4°C to remove the insoluble fraction, and the clarified supernatants were also loaded onto the SDS-PAGE gel. Equal percentage amounts of total cell lysates and total cell culture media were represented on each SDS-PAGE gel. Proteins were transferred onto Immobilon™-P PVDF membranes (Millipore, Billerica, MA). Membranes were blocked with 5% nonfat milk, probed with either a 1:3000 dilution of mouse, anti-V5 antibody, or a 1:3000 dilution of mouse, anti-T7 antibody, and developed by using a 1:1500 dilution of donkey anti-mouse horseradish peroxidase (Jackson ImmunoResearch Laboratories, Inc., #715-035-150). Enhanced Chemiluminescence Plus (Amersham Biosciences, Piscataway, NJ) and a STORM 840 phosphoimager were used to detect protein bands.

Creation of a HeLa cell clone that stably secretes a V5-His epitope tagged CEMIP protein. To purify and characterize the properties of CEMIP protein, we developed a HeLa cell line (clone A11-4) that expresses a compound V5-His epitope-tagged CEMIP under the inducible control of a doxycycline regulated promoter. V5/His-tagged CEMIP was cut out of the pcDNA3.1/V5/His vector with KpnI and PmeI, the overhangs filled in, and blunt-end ligated into the PmeI site of pcDNA4/TO/myc-His (Invitrogen, Carlsbad, CA). T-REx™-HeLa cells were seeded at 12,000 cells/100 mm dish. The next day the cells were transfected with 20 µg of pcDNA4/CEMIP-V5/His plasmid construct using TransIT®-HeLa Monster transfection reagent (Mirus, Madison, WI) following the manufacturer's protocol. Two days after transfection, pcDNA4-containing cells were selected for using 150 µg/ml of zeocin. Stable clones that grew out of the zeocin selection were confirmed for inducible CEMIP expression by treatment with 1 µg/ml doxycycline for 24 h and then assayed by Western blot. The procedure for creating a pool of stable control clones was the same as above except an empty pcDNA4/TO/myc-His vector was used for transfection.

Purification of recombinant CEMIP. T-REx™-HeLa cells expressing the inducible V5/His-tagged CEMIP were seeded into 1700 cm^2 expanded surface roller bottles (Corning Inc., Corning,

NY) and grown to ~75% confluence, whereupon fresh media was added that contained 1 µg/ml doxycycline and low IgG serum. After 72 h, the media was collected and centrifuged for 5 min at 2,000 rpm to remove any cellular debris. The media was then frozen and sent to Roche Protein Expression Group (RPEG) (Roche, Indianapolis, IN) for purification of the V5/His- tagged CEMIP. Briefly, CEMIP was purified by a two-step process which comprised of a Ni-affinity step to bind the 6xHis tag of CEMIP and then a size exclusion chromatography step using a Sephadex G-200 column. Aliquots of the collected fractions were run on an SDS-PAGE gel and analyzed by Coomassie blue staining. The CEMIP-containing fractions were pooled and a concentrated using a Centricon YM-10 centrifugal filter (Millipore, Billerica, MA), and the protein concentration was determined using the Bradford assay. To determine the purity of the CEMIP protein, a sample was run on an SDS-PAGE gel and densitometry was performed using an AlphaImager (Alpha Innotech, San Leandro, CA). Identity of the CEMIP band was confirmed both by mass spectrometry and by western blotting with an anti-6xHis-tag antibody (Roche, #11922416001).

Generation of anti-CEMIP monoclonal antibodies. Generation of anti-CEMIP monoclonal antibodies was performed under contract by Celliance Corporation (Norcross, GA). Briefly, 25 µg of purified recombinant protein was added to 200µl of Ribi or Complete Freund's Adjuvant and then injected sub-cutaneously into female Balb/c mice. Supernatants from the hybridomas were first screened for anti-CEMIP activity by ELISA using purified V5/His-tagged CEMIP. Medias positive by ELISA for anti-CEMIP activity were then further screened for endogenous CEMIP western blot activity using purified T7-tagged CEMIP and FET cell lysates, as well as screened for immunoprecipitation activity using media collected from CEMIP expressing (FET and V411) and non-expressing (V364 and RKO) cell lines. Hybridomas that tested positive for anti-CEMIP activity were injected into mice and the monoclonal antibodies were purified from the ascites using Protein G beads from Roche following the manufacture's protocol.

Ki-67, CD31, CD45, and cleaved caspase-3 immunohistochemistry. The antibodies Ki-67 (Dako, #M7187), Cleaved Caspase-3 (Cell Signaling, #9661), CD31 (Abcam, #ab28364), and CD45 (R&D Systems, #MAB114) were used for immunostaining. Immunostaining was similar as described in the main text for CEMIP except for the following changes. Antigen retrieval was performed by steaming at 98.5°C for 20 min in 10 mM citrate buffer (pH 6.0), nonspecific protein blocking was performed for 20 min, and the antibodies were diluted in Serum-Free Protein Block (Dako). Antibody dilution and incubation times were as follows, Ki-67, 1:50 dilution at room temperature for 1 h, CD31 and CD45, 1:100 dilution at room temperature for 30 min, and cleaved caspase-3, 1:100 dilution at 4ºC overnight. After primary incubation, the slides were washed and Envision ™+ HRP Anti Mouse kit (Ki-67) or Envision ™+ HRP Anti Rabbit kit (CD31 and cleaved caspase-3) (Dako) was used for development, applying secondary antibody conjugated to a polymer-HRP, following manufacturer's protocol. Development times were 5 min (CD31 and cleaved caspase-3) or 10 min (Ki-67). For CD45 detection, after primary incubation the slides were, washed then incubated with an anti-rat secondary antibody (BD Pharmingen, #551013) at a 1:50 dilution for 30 min at room temperature, washed, incubated with Streptaviden-HRP for 30 min at room temperature, washed, and then incubated with substrate-chromogen for 5 min.

SUPPLEMENTARY METHODS REFERENCES

1. Pfaffl MW, Tichopad A, Prgomet C and Neuvians TP. Determination of stable housekeeping genes, differentially regulated target genes and sample integrity: BestKeeper--Excel-based tool using pair-wise correlations. Biotechnol Lett. 2004; 26(6):509-515.
2. Andersen CL, Jensen JL and Orntoft TF. Normalization of real-time quantitative reverse transcription-PCR data: a model-based variance estimation approach to identify genes suited for normalization, applied to bladder and colon cancer data sets. Cancer Res. 2004; 64(15):5245-5250.
3. Hellemans J, Mortier G, De Paepe A, Speleman F and Vandesompele J. qBase relative quantification framework and software for management and automated analysis of real-time quantitative PCR data. Genome Biol. 2007; 8(2):R19.

A.

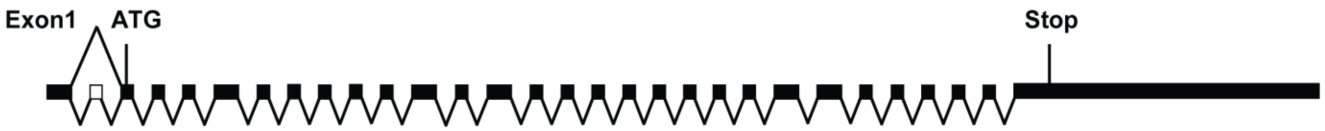

B.
GAGCTAGCGCTCAAGCAGAGCCCAGCGCGGTGCTATCGGACAGAGCCTGGCGAGCGCAAGCGGCGCGGGGAGCCAGCGGGGCTGAGCGCGGCCAGGGTCTGAACCCAGATTTCCCAGACTAGCTACCACTCCGCTTGCCCACGCCCC
GGGAGCTCGCGGCGCCTGGCGGTCAGCGACCAGACGTCCGGGGCCGCTGCGCTCCTGGCCCGCGAGGCGTGACACTGTCTCGGCTACAGACCCAGAG**AGAAAAGCTTCATTCTGGAGGGGAAGGAGTTTTGAGTGCCAAGGATGAAA**
TTCCACCCATCACTCGGTCTCTGAGCTGCAGGACACAGGCAGGACAACGGTAGGATTTTCATGCCCCGATCTGCCTGGCCTTGAGTTGTGGCAGCTGGGGAAGCCACTGGGAGCACACTGCCAGG*<u>ATG</u>*GGAGCTGCTGGGAGGCAGG
ACTTCCTCTTCAAGGCCATGCTGACCATCAGCTGGCTCACTCTGACCTGCTTCCCTGGGGCCACATCCACAGTGGCTGCTGGGTGCCCTGACCAGAGCCCTGAGTTGCAACCCTGGAACCCTGGCCATGACCAAGACCACCATGTGC
ATATCGGCCAGGGCAAGACACTGCTGCTCACCTCTTCTGCCACGGTCTATTCCATCCACATCTCAGAGGGAGGCAAGCTGGTCATTAAAGACCACGACGAGCCGATTGTTTTGCGAACCCGGCACATCCTGATTGACAACGGAGGAG
AGCTGCATGCTGGGAGTGCCCTCTGCCCTTTCCAGGGCAATTTCACCATCATTTTGTATGGAAGGGCTGATGAAGGTATTCAGCCGGATCCTTACTATGGTCTGAAGTACATTGGGGTTGGTAAAGGAGGCGCTCTTGAGTTGCATG
GACAGAAAAAGCTCTCCTGGACATTTCTGAACAAGACCCTTCACCCAGGTGGCATGGCAGAAGGAGGCTATTTTTTTGAAAGGAGCTGGGGCCACCGTGGAGTTATTGTTCATGTCATCGACCCCAAATCAGGCACAGTCATCCATT
CTGACCGGTTTGACACCTATAGATCCAAGAAAGAGAGTGAACGTCTGGTCCAGTATTTGAACGCGGTGCCCGATGGCAGGATCCTTTCTGTTGCAGTGAATGATGAAGGTTCTCGAAATCTGGATGACATGGCCAGGAAGGCGATGA
CCAAATTGGGAAGCAAACACTTCCTGCACCTTGGATTTAGACACCCTTGGAGTTTTCTAACTGTGAAAGGAAATCCATCATCTTCAGTGGAAGACCATATTGAATATCATGGACATCGAGGCTCTGCTGCTGCCCGGGTATTCAAAT
TGTTCCAGACAGAGCATGGCGAATATTTCAATGTTTCTTTGTCCAGTGAGTGGGTTCAAGACGTGGAGTGGACGGAGTGGTTCGATCATGATAAAGTATCTCAGACTAAAGGTGGGGAGAAAATTTCAGACCTCTGGAAAGCTCACC
CAGGAAAAATATGCAATCGTCCCATTGATATACAGGCCACTACAATGGATGGAGTTAACCTCAGCACCGAGGTTGTCTACAAAAAAGGCCAGGATTATAGGTTTGCTTGCTACGACCGGGGCAGAGCCTGCCGGAGCTACCGTGTAC
GGTTCCTCTGTGGGAAGCCTGTGAGGCCCAAACTCACAGTCACCATTGACACCAATGTGAACAGCACCATTCTGAACTTGGAGGATAATGTACAGTCATGGAAACCTGGAGATACCCTGGTCATTGCCAGTACTGATTACTCCATGT
ACCAGGCAGAAGAGTTCCAGGTGCTTCCCTGCAGATCCTGCGCCCCCAACCAGGTCAAAGTGGCAGGGAAACCAATGTACCTGCACATCGGGGAGGAGATAGACGGCGTGGACATGCGGGCGGAGGTTGGGCTTCTGAGCCGGAACA
TCATAGTGATGGGGGAGATGGAGGACAAATGCTACCCCTACAGAAACCACATCTGCAATTTCTTTGACTTCGATACCTTTGGGGGCCACATCAAGTTTGCTCTGGGATTTAAGGCAGCACACTTGGAGGGCACGGAGCTGAAGCATA
TGGGACAGCAGCTGGTGGGTCAGTACCCGATTCACTTCCACCTGGCCGGTGATGTAGACGAAAGGGGAGGTTATGACCCACCCACATACATCAGGGACCTCTCCATCCATCATACATTCTCTCGCTGCGTCACAGTCCATGGCTCCA
ATGGCTTGTTGATCAAGGACGTTGTGGGCTATAACTCTTTGGGCCACTGCTTCTTCACGGAAGATGGGCCGGAGGAACGCAACACTTTTGACCACTGTCTTGGCCTCCTTGTCAAGTCTGGAACCCTCCTCCCCTCGGACCGTGACA
GCAAGATGTGCAAGATGATCACAGAGGACTCCTACCCAGGGTACATCCCCAAGCCCAGGCAAGACTGCAATGCTGTGTCCACCTTCTGGATGGCCAATCCCAACAACAACCTCATCAACTGTGCCGCTGCAGGATCTGAGGAAACTG
GATTTTGGTTTATTTTTCACCACGTACCAACGGGCCCCTCCGTGGGAATGTACTCCCCAGGTTATTCAGAGCACATTCCACTGGGAAAATTCTATAACAACCGAGCACATTCCAACTACCGGGCTGGCATGATCATAGACAACGGAG
TCAAAACCACCGAGGCCTCTGCCAAGGACAAGCGGCCGTTCCTCTCAATCATCTCTGCCAGATACAGCCCTCACCAGGACGCCGACCCGCTGAAGCCCCGGGAGCCGGCCATCATCAGACACTTCATTGCCTACAAGAACCAGGACC
ACGGGGCCTGGCTGCGCGGCGGGGATGTGTGGCTGGACAGCTGCCGGTTTGCTGACAATGGCATTGGCCTGACCCTGGCCAGTGGTGGAACCTTCCCGTATGACGACGGCTCCAAGCAAGAGATAAAGAACAGCTTGTTTGTTGGCG
AGAGTGGCAACGTGGGGACGGAAATGATGGACAATAGGATCTGGGGCCCTGGCGGCTTGGACCATAGCGGAAGGACCCTCCCTATAGGCCAGAATTTTCCAATTAGAGGAATTCAGTTATATGATGGCCCCATCAACATCCAAAACT
GCACTTTCCGAAAGTTTGTGGCCCTGGAGGGCCGGCACACCAGCGCCCTGGCCTTCCGCCTGAATAATGCCTGGCAGAGCTGCCCCCATAACAACGTGACCGGCATTGCCTTTGAGGACGTTCCGATTACTTCCAGAGTGTTCTTCG
GAGAGCCTGGGCCCTGGTTCAACCAGCTGGACATGGATGGGGATAAGACATCTGTGTTCCATGACGTCGACGGCTCCGTGTCCGAGTACCCTGGCTCCTACCTCACGAAGAATGACAACTGGCTGGTCCGGCACCCAGACTGCATCA
ATGTTCCCGACTGGAGAGGGGCCATTTGCAGTGGGTGCTATGCACAGATGTACATTCAAGCCTACAAGACCAGTAACCTGCGAATGAAGATCATCAAGAATGACTTCCCCAGCCACCCTCTTTACCTGGAGGGGGCGCTCACCAGGA
GCACCCATTACCAGCAATACCAACCGGTTGTCACCCTGCAGAAGGGCTACACCATCCACTGGGACCAGACGGCCCCCGCCGAACTCGCCATCTGGCTCATCAACTTCAACAAGGGCGACTGGATCCGAGTGGGGCTCTGCTACCCGC
GAGGCACCACATTCTCCATCCTCTCGGATGTTCACAATCGCCTGCTGAAGCAAACGTCCAAGACGGGCGTCTTCGTGAGGACCTTGCAGATGGACAAAGTGGAGCAGAGCTACCCTGGCAGGAGCCACTACTACTGGGACGAGGACT
CAGGGCTGTTGTTCCTGAAGCTGAAAGCTCAGAACGAGAGAGAGAAGTTTGCTTTCTGCTCCATGAAAGGCTGTGAGAGGATAAAGATTAAAGCTCTGATTCCAAAGAACGCAGGCGTCAGTGACTGCACAGCCACAGCTTACCCCA
AGTTCACCGAGAGGGCTGTCGTAGACGTGCCGATGCCCAAGAAGCTCTTTGGTTCTCAGCTGAAAACAAAGGACCATTTCTTGGAGGTGAAGATGGAGAGTTCCAAGCAGCACTTCTTCCACCTCTGGAACGACTTCGCTTACATTG
AAGTGGATGGGAAGAAGTACCCCAGTTCGGAGGATGGCATCCAGGTGGTGGTGATTGACGGGAACCAAGGGCGCGTGGTGAGCCACACGAGCTTCAGGAACTCCATTCTGCAAGGCATACCATGGCAGCTTTTCAACTATGTGGCGA
CCATCCCTGACAATTCCATAGTGCTTATGGCATCAAAGGGAAGATACGTCTCCAGAGGCCCATGGACCAGAGTGCTGGAAAAGCTTGGGGCAGACAGGGGTCTCAAGTTGAAAGAGCAAATGGCATTCGTTGGCTTCAAAGGCAGCT
TCCGGCCCATCTGGGTGACACTGGACACTGAGGATCACAAAGCCAAAATCTTCCAAGTTGTGCCCATCCCTGTGGTGAAGAAGAAGAAGTTG***<u>TGA</u>***

C.
MGAAGRQDFLFKAMLTISWLTLTCFPGATSTVAAGCPDQSPELQPWNPGHDQDHHVHIGQGKTLLLTSSATVYSIHISEGGKLVIKDHDEPIVLRTRHILIDNGGELHAGSALCPFQGNFTIILYGRADEGIQPDPYYGLKYIGVGK
GGALELHGQKKLSWTFLNKTLHPGGMAEGGYFFERSWGHRGVIVHVIDPKSGTVIHSDRFDTYRSKKESERLVQYLNAVPDGRILSVAVNDEGSRNLDDMARKAMTKLGSKHFLHLGFRHPWSFLTVKGNPSSSVEDHIEYHGHRGS
AAARVFKLFQTEHGEYFNVSLSSEWVQDVEWTEWFDHDKVSQTKGGEKISDLWKAHPGKICNRPIDIQATTMDGVNLSTEVVYKKGQDYRFACYDRGRACRSYRVRFLCGKPVRPKLTVTIDTNVNSTILNLEDNVQSWKPGDTLVI
ASTDYSMYQAEEFQVLPCRSCAPNQVKVAGKPMYLHIGEEIDGVDMRAEVGLLSRNIIVMGEMEDKCYPYRNHICNFFDFDTFGGHIKFALGFKAAHLEGTELKHMGQQLVGQYPIHFHLAGDVDERGGYDPPTYIRDLSIHHTFSR
CVTVHGSNGLLIKDVVGYNSLGHCFFTEDGPEERNTFDHCLGLLVKSGTLLPSDRDSKMCKMITEDSYPGYIPKPRQDCNAVSTFWMANPNNNLINCAAAGSEETGFWFIFHHVPTGPSVGMYSPGYSEHIPLGKFYNNRAHSNYRA
GMIIDNGVKTTEASAKDKRPFLSIISARYSPHQDADPLKPREPAIIRHFIAYKNQDHGAWLRGGDVWLDSCRFADNGIGLTLASGGTFPYDDGSKQEIKNSLFVGESGNVGTEMMDNRIWGPGGLDHSGRTLPIGQNFPIRGIQLYD
GPINIQNCTFRKFVALEGRHTSALAFRLNNAWQSCPHNNVTGIAFEDVPITSRVFFGEPGPWFNQLDMDGDKTSVFHDVDGSVSEYPGSYLTKNDNWLVRHPDCINVPDWRGAICSGCYAQMYIQAYKTSNLRMKIIKNDFPSHPLY
LEGALTRSTHYQQYQPVVTLQKGYTIHWDQTAPAELAIWLINFNKGDWIRVGLCYPRGTTFSILSDVHNRLLKQTSKTGVFVRTLQMDKVEQSYPGRSHYYWDEDSGLLFLKLKAQNEREKFAFCSMKGCERIKIKALIPKNAGVSD
CTATAYPKFTERAVVDVPMPKKLFGSQLKTKDHFLEVKMESSKQHFFHLWNDFAYIEVDGKKYPSSEDGIQVVVIDGNQGRVVSHTSFRNSILQGIPWQLFNYVATIPDNSIVLMASKGRYVSRGPWTRVLEKLGADRGLKLKEQMA
FVGFKGSFRPIWVTLDTEDHKAKIFQVVPIPVVKKKKL

Supplementary Figure 1. Structure of the *CEMIP* gene. **(A).** Black boxes denote *CEMIP* gene exons comprising the short *CEMIP* transcript, with the white box denoting the additional exon included in the longer form of the transcript. **(B).** Nucleotide sequence of the *CEMIP* coding region, with the start and stop codons in *italics* and *underlined*, and the exon in the long form of the transcript in bold. **(C).** Amino acid sequence specified by the *CEMIP* coding region.

A.

B.

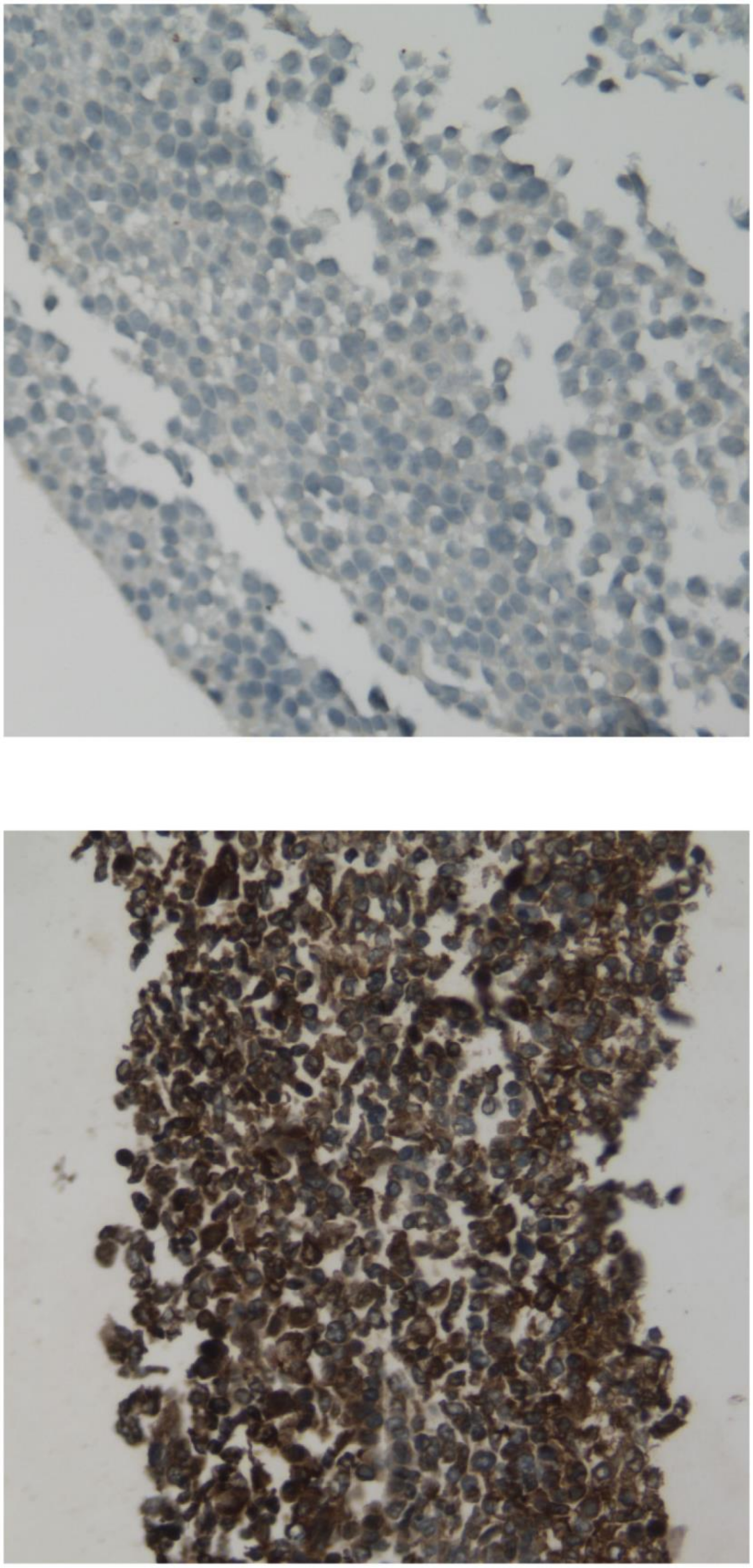

Supplementary Figure 2. Immunohistochemistry of formalin fixed parafiin embedded cell pellets from non-expressing RKO colon cancer cells **(A)**, and CEMIP transcript expressing FET colon cancer cells **(B)** using anti-CEMIP monoclonal antibody PW-3.

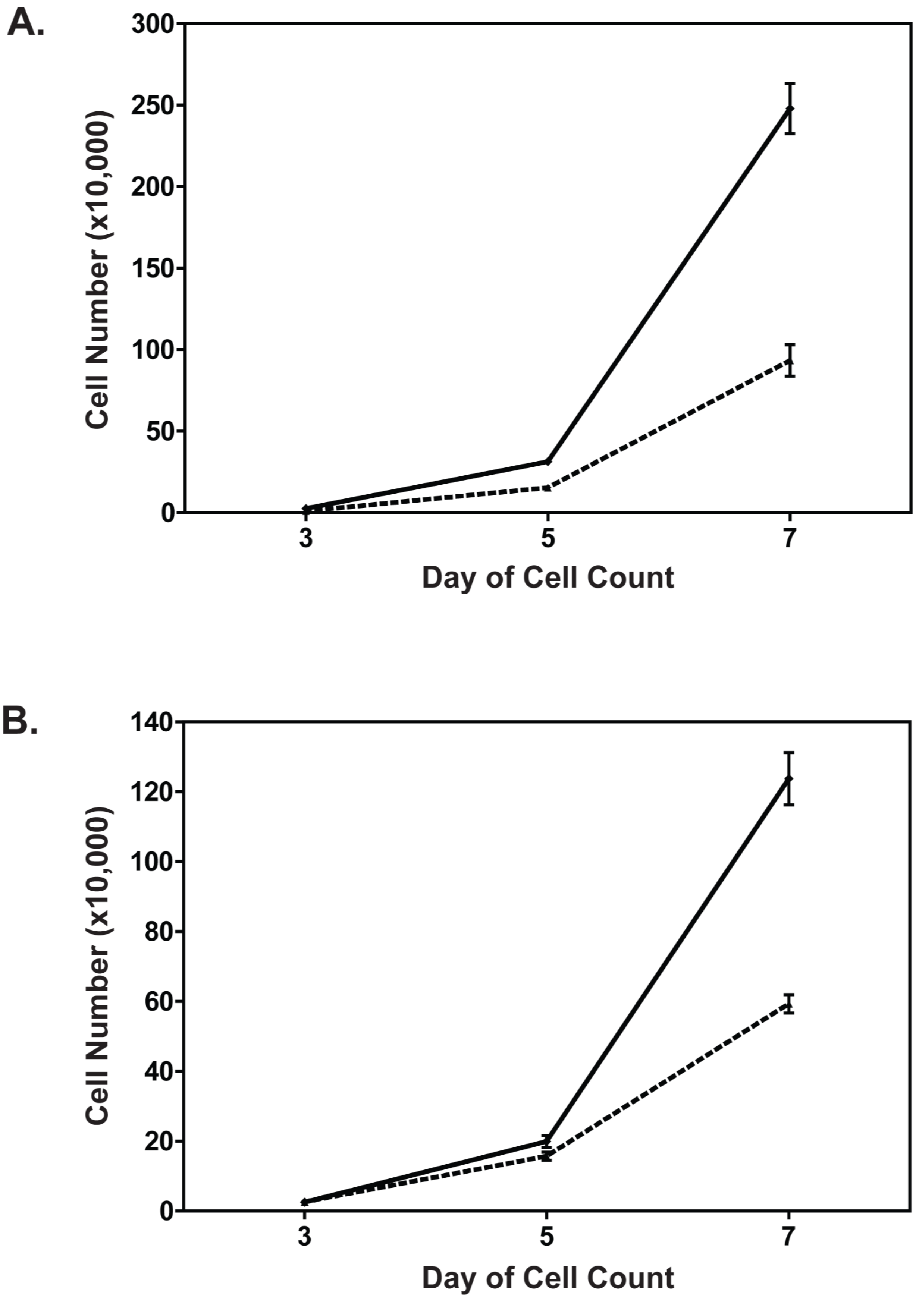

Supplementary Figure 3. Growth curves for *CEMIP* deleted DLD-1 clones (dashed line) as compared to wild-type DLD-1 (solid line) for knockout Clone A **(A)**, and knockout Clone B **(B)**. Error bars are standard errors of the mean.

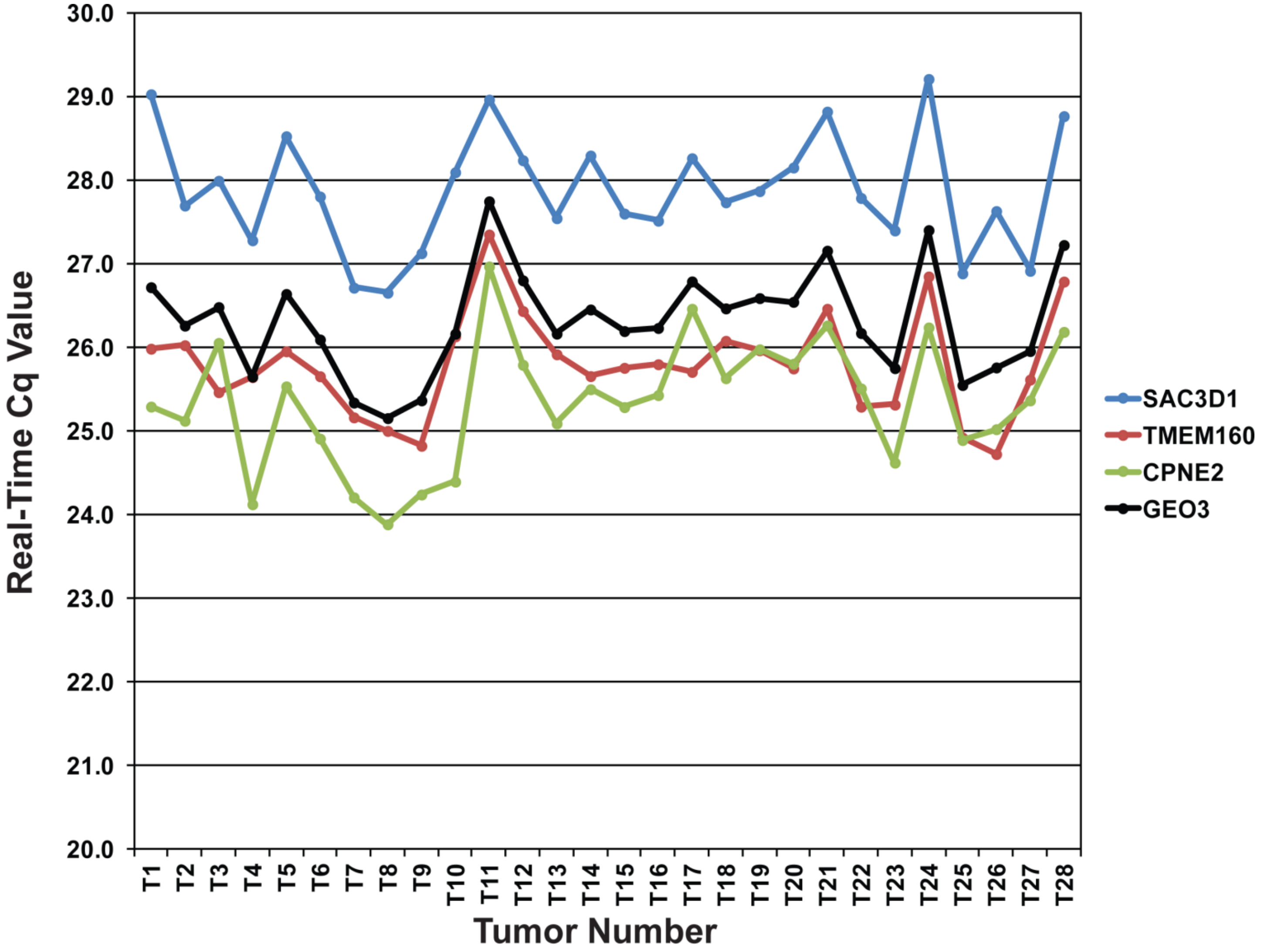

Supplementary Figure 4. Cq values for individual SAC3D1, TMEM160, and CPNE2 transcripts determined in 1 µg of input total RNA across a panel of 28 colon tumor samples. The geometric mean of the Cq values (GEO3) for all three genes is also plotted. Note the y-axis scale begins at Cq = 20.

Supplementary Table S1. Comparison of the top four most stable transcripts as determined by three different software programs used for determining optimal normalization genes for real-time PCR studies.

Program	RANK			
	First	Second	Third	Fourth
Normfinder	CPNE2	ZNF787	SIRT3	TMEM160
BestKeeper	TMEM134	TMEM160	SAC3D1	CPNE2
genorm^{PLUS}	SAC3D1	TMEM160	CPNE2	DDA1
